# Improving Treatment Together: a protocol for a multi-phase, community-based participatory, and co-design project to improve youth opioid treatment service experiences in British Columbia

**DOI:** 10.1186/s13722-021-00261-7

**Published:** 2021-08-14

**Authors:** Kirsten Marchand, Corinne Tallon, Christina Katan, Jill Fairbank, Oonagh Fogarty, Katrina Marie Pellatt, Roxanne Turuba, Steve Mathias, Skye Barbic

**Affiliations:** 1 Foundry, 915-1045 Howe Street, Vancouver, BC V6Z 2A9 Canada; 2 Faculty of Medicine, University of British Columbia, 317-2194 Health Sciences Mall, Vancouver, BC V6T 1Z3 Canada; 3 Department of Occupational Science and Occupational Therapy, University of British Columbia, 317-2194 Health Sciences Mall, Vancouver, BC V6T 1Z3 Canada; 4 Canadian Centre on Substance Use and Addiction, 75 Albert St #500, Ottawa, ON K1P 5E7 Canada; 5 Centre for Health Evaluation Outcome Sciences, 588-1081 Burrard Street, Vancouver, BC V6Z 1Y6 Canada; 6 Providence Health Care, St. Paul’s Hospital, Vancouver, BC V6Z 1Y6 Canada; 7 Providence Health Care Research Institute, Vancouver, BC V6Z 1Y6 Canada

**Keywords:** Opioid use, Opioid use disorder, Youth, Adolescents, Young adults, Human-centred design, Co-design, Community-based participatory research, Implementation science

## Abstract

**Background:**

Opioid use is one of the most critical public health issues as highly potent opioids contribute to rising rates of accidental opioid-related toxicity deaths. This crisis has affected people from all age groups, including youth (ages 15–24) who are in a critical developmental period where the stakes of opioid use are especially high. Efforts to reduce the significant harms of opioid use have focused on the expansion of evidence-based treatments, including medications for opioid use disorder (e.g. buprenorphine). While these treatments are unequivocally life saving, recent evidence suggests that they may not align with youths’ needs. Accordingly, the ‘Improving Treatment Together’ (ITT) project has been designed with the aim to improve youths’ opioid treatment service experiences and outcomes by co-developing, implementing, and measuring youth-centred opioid use treatment service innovations. This manuscript describes the protocol for this multi-phase project.

**Methods:**

The ITT project follows community-based participatory research (CBPR) and strategically integrates co-design processes throughout its four phases. Upon establishing a project partnership between national, provincial and community-based organizations, Phase 1 follows four core elements of human-centred co-design (empathy, needs identification, ideation, prototyping) in nine separate workshops. These workshops will be held in four diverse communities with youth, caregivers and service providers who have accessed or delivered opioid treatment services. Phase 1 will culminate in the co-production of opioid treatment service innovations to be considered by the project’s partners for further co-development, pilot testing, and wider implementation during the remaining phases of the project. Throughout each phase, the project will collect and analyse both qualitative and quantitative research and evaluation data to determine the project’s impact.

**Discussion:**

This protocol provides a detailed description of the ITT project, with an emphasis on the project’s application of co-design and CBPR processes, the planned research and implementation procedures, and the establishment of a unique partnership. To our knowledge, this is one of the first projects to integrate these participatory processes to the design, implementation and measurement of youth-centred opioid treatment services. Embedding these processes throughout each phase of the project will strengthen the relevance and feasibility of the project’s service delivery innovations.

**Supplementary Information:**

The online version contains supplementary material available at 10.1186/s13722-021-00261-7.

## Introduction

Opioids, such as morphine, are a class of essential medicines that have been used for centuries in the treatment of pain (e.g. post-operative, palliative) [1]. Today, opioid use has become one of the most critical public health issues as non-medical prescription opioid use, illicit opioid use (e.g. heroin), and opioid use disorder (OUD) have increased steadily over the last three decades [2–5]. The urgency of this issue is most apparent in North America, where highly potent opioids (mainly fentanyl and its analogues) have contributed to rising rates of non-fatal and fatal opioid-related drug toxicity events [4, 6–8]. In Canada, a total of 19,355 people have experienced a fatal opioid-related drug toxicity event since 2016, and the 2020 age-adjusted death rate was 16.1 per 100,000 [9]. While the incidence of these fatal outcomes has been highest for adults [10], adolescents and young adults (ages 15–24, collectively referred to as “youth”) have consistently accounted for approximately 20% of these preventable deaths [10, 11]. Youth have also experienced the fastest growing rate of opioid toxicity-related hospitalizations compared to adults [12].

The stakes of opioid use during the current crisis are particularly high for youth. This is a critical developmental period, and an age period when substance use initiation is at its peak [13–16]. Research into perceptions of substance use suggest that youth view it as acceptable and with low risks; and thus, youth may be less likely to recognize the harms associated with substance use and when support may be needed [15, 17–19]. It is also widely known that earlier initiation of opioid use increases the risk of developing OUD and other longer-term health and social harms (e.g. poly-substance use disorders, blood-borne infections, legal problems) [20–22].

Accordingly, understanding and responding to opioid use and OUD among youth necessitates an approach that considers these unique patterns, risk factors, and harms [14, 15]. Recent clinical practice guidelines have recommended that service providers be able to offer youth multiple approaches, including early intervention, psychosocial treatment (e.g. cognitive behavioural therapy), and medications for opioid use disorder (MOUD; e.g. buprenorphine) in the context of recovery-oriented, harm reduction, and youth-centred frameworks [14]. Similar to adult populations with OUD, clinical practice guidelines and recent reviews recommend MOUD as a first-line treatment for youth [14, 23–25].

However, the effectiveness of this range of interventions remains largely understudied among youth using opioids [14, 23, 24]. Emerging evidence also suggests that youth encounter barriers to seeking and remaining in opioid treatment services [26–28]. For instance, in the context of MOUD, recent studies have shown that youth experience barriers at individual (e.g. gender-identity; treatment preferences), interpersonal (e.g. family support; stigma), and institutional-levels (e.g. age-based policies; wait times; prescriber availability) [26–28]. These barriers may support explanation of the relatively low rate of MOUD uptake and long-term engagement among youth [15, 29–31]. In the Canadian context for example, a recent population-level study has shown that children and youth (ages 10–24) were less likely than older age groups to have initiated MOUD in the 30-days following a non-fatal opioid-related drug toxicity event [32]. Collectively, these findings stress the need for further research into youths’ barriers to opioid use treatments and opportunities to develop and implement youth-centred approaches.

In order to understand the full range of these barriers and opportunities, this research may benefit from a multi-stakeholder approach that meaningfully and equitably engages youth who use opioids, their caregivers, service providers, researchers, and decision-makers. Indeed, the meaningful engagement of people with lived/living substance use experience is recommended for both youth- and substance use-focused research [33–37]. Community-based participatory research (CBPR) is one such approach that embeds authentic, collaborative and equitable partnerships into the research process and aims to reduce health disparities through social change or action [38, 39]. Co-design processes share these values and are increasingly applied in healthcare and public policy to develop actionable, innovative, and effective solutions to complex problems [40, 41]. Therefore, both CBPR and co-design are particularly suited to the challenge of developing and implementing youth-centred opioid treatments.

Using these methods, the overarching aim of the ongoing multi-phase ‘Improving Treatment Together (ITT)’ project is to improve youths’ opioid treatment service experiences. As a multi-phase project, the objectives are specific to each phase, and are as follows. Phase 1 aims to: (a) understand the opioid treatment service experiences and needs of youth, parents/caregivers, and service providers; and (b) to brainstorm youth-centred solutions to be considered for future development, implementation and measurement. Phase 2 aims to: (a) select and co-design youth-centred opioid treatment service prototypes to improve opioid treatment experiences and outcomes. Phase 3 aims to: (a) implement the co-designed prototypes in the local community; and (b) evaluate the prototype’s impact on opioid treatment service experiences and outcomes. Lastly, Phase 4 aims to: (a) refine the co-designed prototypes with community-based partners; and (b) co-develop long-term plans for sustaining the prototype locally, wider scaling, and measurement.

To our knowledge, this is one of the first projects to combine CBPR and co-design methods to address youth-centred opioid treatment service innovations. By combining these methods, we will contribute evidence that supports the development of innovations that may improve the opioid treatment service experiences and outcomes of youth using opioids. These methods are highly iterative as they adapt to the preferences and needs of participants, communities, and other important stakeholders [41]. Thus, the objective of this manuscript is to document the initial protocol developed for the ITT project to serve as both a record for reporting project outcomes and an example for others considering the combination of these complementary approaches in substance use research, practice and policy.

## Methods

### Overarching project design

The ITT project uniquely integrates CBPR and co-design approaches to produce actionable evidence leading to the development, implementation and measurement of youth-centred opioid treatment service innovations. CBPR is an orientation to research that lies between action research and participatory research traditions. It is based on a set of core principles, including co-learning, community capacity building, equal benefits, and a long-term commitment to reduce health disparities [38, 39]. Co-design has its roots in the human-centered design field and has been increasingly adapted to healthcare, with the aim of improving healthcare experiences and outcomes by co-designing health services, interventions, or innovative products with patients, caregivers and healthcare service providers [41, 42]. CBPR and co-design are complementary in their emphasis on co-creation, engagement of “end users” (i.e. people with lived/living experience, caregivers, service providers), and systematic and iterative processes [41, 43]. There are also important differences between these approaches. Notably, the co-design process tends to be shorter in timeline with a focus on prototype development, while CBPR tends to be longer, focused on community relationships, and production of research for future action [41].

**Fig. 1:**
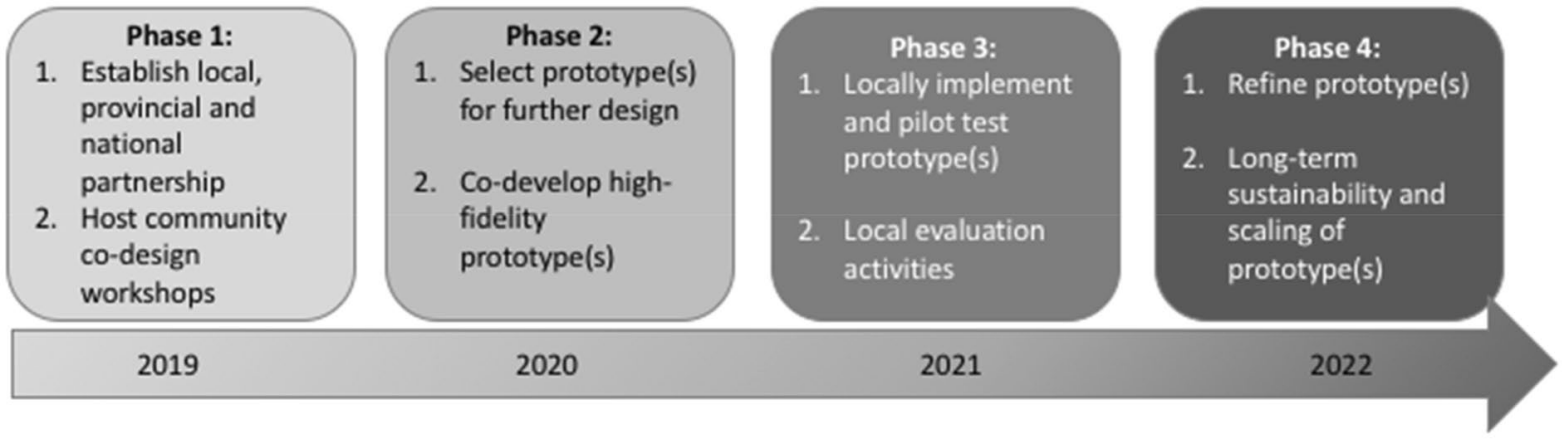
Timeline of major activities for each phase of the Improving Treatment Together project

The ITT project weaves together the complementary features of CBPR and co-design throughout its four phases over its 4-year period (Fig. 1). In effort to follow best practices for the reporting of CBPR [44], the anticipated CBPR activities, outcomes and outputs are outlined by project phase in Table 1 and described in detail throughout subsequent sections. Briefly, Phase 1 will establish partnerships with local communities, a project operational and governance structure (including project team capacity, workloads, and roles), and engage community partners in the development of the research protocol and carry out co-design workshops. Phase 2–4 will co-develop, locally test, and evaluate the youth-centered opioid service innovations following implementation science frameworks [45, 46]. As is common to both CBPR and co-design [41], the project will collect and analyse both qualitative and quantitative data to support insight into participants’ experiences and broader interpretation and generalization of the findings.

**Table 1:** Summary of planned community-based participatory research activities at each project phase

Phase	Planned CBPR activities and techniques	Anticipated outcomes and outputs
1	National, provincial, and community-based partnerships and operational structure established^1^ Core project team, roles, workloads, and communication processes established across each partner^2^ Training of project team members in CBPR and co-design processes Project team carries out community-based co-design workshops Project team leads multi-site thematic analyses of workshop data, involving community-partners and peer researchers at each stage of analysis Project team leads process evaluation	Within and across stakeholder findings Knowledge translation activities (i.e. reports, scientific publications, presentations) Evaluation of community-based co-design workshop process
2	Project team to synthesize main findings of Phase 1 and prototype summary in close collaboration with project partners at key decision points All project partners come together for prototype selection process Capacity assessments with local partners to create prototype-specific operational structure Prototype-specific working groups established and co-develops high-fidelity prototype, its purpose, content, and anticipated outcomes^3^	Summary of low-fidelity prototypes Selection and development of 2–3 high-fidelity prototypes
3	Prototype specific working groups make local implementation plan Working group locally implement prototype(s) with prototype-naïve population(s) Provincial evaluation team leads prototype-specific process and outcome evaluation	Reports and presentations of process and outcome evaluation findings for each prototype
4	National, provincial, and community-partner meeting to discuss key findings from Phase 3 to be prepared for wider dissemination (i.e. policy and academic audiences) National, provincial, and community-partner meeting to discuss opportunities for wider scaling National, provincial, and community-partner meeting to discuss new research questions and	Knowledge summaries, academic publications, presentations of Phase 3 New funding proposals for wider implementation and

CBPR: community-based participatory research

^1^National organization—Canadian Centre on Substance Use and Addiction (CCSA); Provincial organization—Foundry Central Office (FCO); Local organizations—community-based Foundry Centres

^2^Core project team comprising of one or more members from across all three partners, including researchers, evaluation specialists, knowledge brokers, and youth and family peer team members

^3^Prototype-specific working group structure will depend on the capacity assessment. It will aim to include participant representatives (youth, parents/caregivers, service providers), implementation champions from the local community Centres, and the project team (youth/family peer research team members, researchers, evaluation associates, knowledge brokers)

### Study setting, participants, and recruitment

The project is taking place in the province of British Columbia (BC), Canada, which has the highest national rate of fatal and non-fatal opioid-related drug toxicity events [9]. Aligning with the project’s CBPR approach, a partnership has been established between national, provincial, and community-based organizations: (1) the Canadian Centre on Substance Use and Addiction (CCSA), a national non-governmental organization providing leadership on solutions to address substance-related harms [47]; (2) Foundry Central Office, the provincial backbone team for a network of community-based centres delivering integrated youth services for mental health, substance use and primary care [48, 49]; and (3) four community-based Foundry centres offering MOUD (Vancouver, Victoria, Kelowna, Prince George).

With the support of each community-based Foundry centre, we plan to purposively sample participants representing three stakeholder groups: (1) youth with lived/living opioid use experience; (2) parents/caregivers of youth with lived/living opioid use experience; and (3) service providers delivering opioid use services to youth. To be eligible, all participants will: (a) be able to speak and write in English; and (b) be willing and able to provide fully informed consent to participate. Additional criteria have been specified for each participant group. Youth participants will be eligible if they: (a) are between the ages of 16–24; (b) have current or recent (12-months) experience using opioids (other than as prescribed by a medical professional); and (c) have current or recent (12-months) experience accessing/receiving health services or treatment for opioid use. Parent/caregiver participants will be eligible if they self-identify as a biological parent, adopted parent, step-parent, or legal/non-legal guardian to a youth ages 16–24 who meets the above criteria [50]. Finally, service provider participants will be eligible if they are: (a) a mental health and substance use professional; and (b) provide substance use treatment services (including for opioid use) to youth between the ages of 16–24 years.

Participants from each group will be recruited with the support of the collaborating community-based Foundry Centre, which also include youth and family peer members who have lived/living experience with youth opioid use and opioid treatment services. Centres will be asked to display and distribute recruitment materials that include information about the study, eligibility criteria and the team’s contact information. Interested participants will then contact the study team to review the self-reported eligibility criteria and receive further information.

### Phase 1: co-design workshop procedures

We plan to conduct nine workshops across the four partnering communities. By participant group, the planned workshops will be for: youth (n = 2 workshops in Vancouver and Kelowna); parents/caregivers (n = 3 workshops in Vancouver, Victoria, and Prince George); and service providers (n = 4 workshops in each community). We will conduct separate workshops for each participant group (e.g. youth only) and aim to reach approximately ten participants in each, for a maximum of 90 participants across all workshops. Phase 1 workshops will move through the core co-design elements (Additional file 1) [51]. These elements, their purpose and planned workshop activities are outlined in Table 2. Four members of the project team will facilitate the workshops; including Youth/Family Team Members with lived/living opioid use experience, Research Coordinators, and Knowledge Brokers. All team members have undertaken extensive training in co-design, CBPR, and workshop/focus group facilitation methods.

**Table 2:** Co-design activities for Phase 1 discovery and design workshops

Co-design element	Purpose	Activities
1. Empathizing	Understand the end user experience	Independent reflection using Empathy and Journey Maps Small and large group discussion
2. Identifying needs	Articulate and prioritize the root problems or needs based on the end user experience	Independent brainstorm of needs Small and large group discussion of needs and their prioritization
3. Ideating	Brainstorm solutions that will respond to the prioritized needs	Small and large group discussion
4. Rapid prototyping	Rapidly design low-fidelity prototypes to be considered for future development in the real-world context	Pitch activity Small and large group discussion

Upon arrival to the workshop, participants will review and sign the informed consent form and be asked to respond to an optional brief stakeholder-specific anonymous questionnaire focused on demographics (e.g. age, gender identity, ethnicity, education), substance use (e.g. types of substances used, frequency of use) and healthcare experiences (e.g. treatment/service types treatment settings). From here, the facilitators will open the workshop with introductions and a community agreement for the co-creation of a safe space. The main activities of the workshop will be divided into two carefully guided sessions, “Discovery” and “Design”, with each lasting approximately 3.5 h (see "Discovery session" section for further session details). All activities of the workshop will be audio-recorded, and project team members will take extensive field notes throughout. At the end of the workshop, participants will be asked to complete a voluntary and anonymous feedback survey for the co-design process evaluation.

Participant’s safety and comfort will be of utmost priority throughout the course of these workshops. Workshops will be held in a centrally located venue that is safe and convenient. Participants will be provided with an honorarium for their time ($60–$200 depending on time in the workshop), meals/snacks, and reimbursement for transportation costs. In addition to regularly scheduled breaks, participants will be encouraged to take their own self-care breaks as needed in designated quiet (“chill”) rooms. Peer support will be available through the community partners for all youth and parent/caregiver workshops. Project team members will also be trained in overdose prevention and response, will have naloxone on site, and have resources to support participants in the case of an emergency. These procedures will generally be the same across each of the participant groups and communities.

#### Discovery session

The “Discovery” session is aimed at the first two elements of the co-design process—developing empathy and defining needs. This session will begin with an independent activity guided with tools that build a shared understanding of participants’ journeys accessing or delivering opioid treatment services. These tools will include Journey Maps [52] and Empathy Maps [53] (Additional file 1). Both tools will encourage participants to put themselves at the center of point-of-care interactions and describe their experiences. In the Empathy Map for instance, participants will describe in first-person what they see, think, feel, do, hear, and say. After completing these tools, facilitated small and large group discussions will occur around the positive and difficult experiences of accessing or delivering youth opioid use treatment services. This will support the subsequent co-design element of defining needs and preferences for opioid use treatment services. During this element, participants will independently brainstorm specific needs for improving opioid treatment experiences and outcomes. These needs will then be discussed, grouped and prioritized in small groups.

The small and large group discussions will provide an opportunity to compare and synthesize participants’ experiences and needs. The project team will facilitate these discussions through verbal prompts and clarifying questions, as well as visual aids (i.e. worksheets, post-it notes, flip charts, white board). This session will culminate in a shared understanding of participants’ needs for youth-centered opioid use treatment services.

#### Design session

The “Design” session will build on the prioritization of needs through the second two elements of the co-design process—ideation and rapid prototyping. The guiding question of this session will be *“what could be done to improve the experiences and outcomes of opioid use treatment services for youth?”*. During the ideation element, participants will engage in a facilitated small group brainstorming activity where they will be asked to rapidly brainstorm as many solutions as possible to address the prioritized needs. From here, participants will analyze their ideas, group similar ideas together, and discuss their impacts and implications. Each small discussion group will then select one idea to move forward to the rapid prototyping element. In considering which idea to carry forward, participants will be asked to consider the feasibility and significance of this solution in their community.

These discussions will support the final rapid prototyping element of the workshop, where participants will self-select into small groups to co-design the idea they resonate with the most. During this exercise, the facilitator will guide participants to identify the main problem being addressed by this solution, and the details of a low-fidelity prototype (e.g. how it addresses the problem, for whom it should be offered, when, where, etc.) (Additional file 1). Afterwards, as part of the design thinking process, participants will be asked to bring their low-fidelity prototype to life (e.g. an infographic, a skit, storyboard, etc.) using provided materials. The goal for this activity is to create an interactive and tangible solution that participants can then “test” by obtaining feedback from the other participants. The session will conclude with a large group discussion of the low-fidelity prototypes, where participants will “pitch” their solutions to each other using the provided worksheet (Additional file 1) and provide feedback to refine the ideas.

### Phases 2–4 procedures: prototype development, implementation, and measurement

#### Phase 2: prototype selection and development

As an iterative CBPR and co-design project, the specific procedures for Phases 2–4 will be informed by the findings of Phase 1 (i.e. the low-fidelity prototypes) and the ongoing priorities of the project’s community-based partners. Phase 2 will begin with an extensive prototype selection process. This process will be initiated by the project team and will involve summaries and comparisons of the low-fidelity prototypes, and an environmental scan to ensure that the prototypes have not been developed elsewhere. The project team will engage community-based partners in a series of decision-making meetings, guided by the prototype selection rubric to decide which low-fidelity prototype(s) will move forward to co-development in Phase 2. This rubric considers the prototype’s potential impact, novelty, organizational match, and active implementation timeframe (Additional file 2).

After selecting the prototypes, smaller community-based working groups will be established, depending on the nature of the prototype and partners’ preferences and workloads. The working groups will include participant representatives (youth, parents/caregivers, service providers), implementation specialists from the local community Centres, and the project team (Youth/Family Peer Research Team Members, Researchers, Evaluation Associates, Knowledge Brokers). The working groups will co-develop the high-fidelity prototype(s), including its purpose, expected outcomes, content, and selection of and collaboration with the external vendors (if required). These working groups will remain in place throughout Phase 3 (local implementation and testing) and Phase 4 (prototype refinement and scaling out) as they hold expertise about the barriers and facilitators to the prototype’s implementation (e.g. centre capacity, data infrastructure) [45, 46].

#### Phase 3: local implementation and evaluation of prototypes

During Phase 3, the working groups will develop the local implementation plans of the high-fidelity prototype(s), following implementation science frameworks [45, 46]. Local implementation planning will consider factors such as the target population’s preferences and needs, and organizational barriers and facilitators. From here, the project team will locally implement and pilot test the prototype(s) with prototype-naïve populations in collaboration with the community-based Foundry Centre. The definition and planned sample size of the naïve population will depend on the nature of the prototype(s), and will generally follow the criteria outlined for the Phase 1 co-design workshops (e.g. youth using opioids and accessing opioid treatment services).

Phase 3 will involve research and evaluation of the prototype(s) implementation process and outcomes over a period of 4–6 months. For instance, the evaluation of local implementation will address the extent to which local implementation process occurred as intended. Additionally, research and evaluation questions will be integrated to determine whether the prototype(s) have achieved their intended short-term outcomes. Data collection methods will follow the implementation science frameworks and rely on feedback surveys, case studies, and qualitative interviews/focus groups with the prototype(s) working group and naïve populations. The evaluation will be led by associates at the provincial FCO organization upon agreement by all partners on the evaluation plan. Findings from the evaluation will be shared in reports and presentations with the project team, partners at all levels, and project funders.

#### Phase 4: refine prototypes and plan long-term sustainability and measurement

Findings of the process and outcome evaluation of Phase 3 will be used to guide the working groups’ further refinement of the prototype(s) content and outcomes and to develop plans for long-term sustainability within the local community-based Foundry Centre. This phase will also involve a series of meetings where all project partners will discuss key findings from Phase 3 to determine opportunities for wider dissemination of the prototype(s), scaling, and new research questions and designs (e.g. impact of prototype on opioid use treatment service engagement, satisfaction). This phase will culminate in new funding proposals to provincial/federal health policy organizations and/or research granting agencies.

### Data management and analysis

Table 3 summarizes the main sources of data and planned analyses for Phase 1 and 3, when research and evaluation data will be collected. For both phases, descriptive analyses (e.g. tabular and graphical summaries of means, standard deviations, frequencies) will summarize demographic data and feedback surveys; these analyses will use disaggregated descriptions (e.g. by gender or location) where possible (i.e. for phase 3 pilot test, where sample sizes will be larger and can preserve anonymity). Qualitative data will be transcribed verbatim and thematically analyzed with the goal of developing a rich overall description of participants’ experiences, needs and ideas [54, 55]. As there is limited existing research (particularly among parents/caregivers and service providers), an inductive approach will be applied to the research analyses for Phase 1 and 3. After a careful reading of the data, a semantic and data driven approach will be used to generate initial codes and to search, review and define themes [54]. To facilitate interpretative analyses, we may integrate the quantitative survey data and qualitative themes using matrices and joint displays (e.g. explore themes by community). Quantitative data will be entered and stored to a secure electronic database, and analyzed using Stata, and qualitative data will be managed and analyzed using NVivo. All data will be de-identified, reported in aggregate format and carefully reviewed to preserve participant anonymity.

**Table 3:** Summary of anticipated data sources and analyses plans at each project phase

Phase	Data sources	Analysis plans
1	Research data Demographic surveys Transcripts Worksheets Flip charts	Led by provincial research team members with partner involvement at key decision points (mutual agreement on analysis plan, discuss preliminary and final results, display and interpretation of findings) Descriptive analysis (tabular and graphical summaries) of demographic data Thematic analysis of transcripts and workshop documents Narrative and tabular summaries of all low-fidelity prototypes
	Process evaluation data Feedback surveys Qualitative interviews	Led by provincial evaluation associates with partner involvement at key decision points (mutual agreement on data sources, evaluation plan, discussion of findings and interpretation) Descriptive analysis of feedback surveys Thematic analysis of interview data
2	Research and evaluation data are not collected at this phase	
3	Research and evaluation data Feedback surveys (including demographic data) Case studies Qualitative interviews and focus groups	The provincial research and evaluation teams will work closely to lead data collection and analysis with partner involvement at key decision points (mutual agreement on research and evaluation questions, data collection, discussion of findings and interpretation) Research questions will focus on satisfaction and prototype-specific outcomes associated with local testing Descriptive analysis of feedback surveys Narrative summary of case studies Thematic analysis interviews and focus group data
4	Research and evaluation data are not collected at this phase	

As a CBPR project, the project team will assess all partners’ capacity to engage in the analyses, and will further refine the analysis plan and key decision-points for engagement. The project team and partners will have regular meetings throughout data analysis to support the trustworthiness of the analyses [55], interpretation, and dissemination planning.

### Ethics and dissemination

As this study spans four communities in BC, it requires institutional behavioural research ethics through the harmonized research ethics review and approval process, which has been received through the University of British Columbia/Providence Health Care (Study ID: H19-02077). Phase 1 workshops were completed in February 2020, just before the emergence of the global COVID-19 pandemic. The pandemic has since posed challenges to community-based partner engagement and thus, Phase 1 data analysis and dissemination activities have been delayed while partners have pivoted their attention to other service delivery priorities. Phase 2 activities are in-progress, and Phases 2–4 will be ongoing until September 2022.

As a CBPR project, our dissemination strategy has first considered the knowledge product needs of our community-based partners. Accordingly, community-specific reports, knowledge summaries, and presentations will be the main knowledge products used to summarize project findings. The ITT project also fills critical gaps in the scientific literature concerning youth-centred opioid use treatment experiences, needs, and opportunities. We will also prepare scientific conference presentations, abstracts and peer-reviewed manuscripts to mobilize these findings more widely. All knowledge products will follow best practices for reporting CBPR [44].

## Discussion

This manuscript describes the protocol for the Improving Treatment Together project, which is widely aimed at improving the experiences and outcomes of youth opioid use treatment services. To meet these goals, the project uniquely integrates the complementary features of community-based participatory research and co-design. As methods of engagement are increasingly recommended in youth-focused and substance use research [33–37], the protocol serves as a timely example for others considering the integration and application of these methods in similar settings.

Following the principles of CBPR [38, 39], the first step of this project focused on creating partnerships between national, provincial, and community-based organizations that are united in their mission to ensure that youth have access to evidence-based substance use treatment services. Nationally, CCSA provides leadership on such innovative and evidence-informed approaches with the aim of reducing knowledge to practice gaps. Provincially, FCO is a leader in the transformation of youth and family-centred mental health and substance use service integration and delivery. Together, and more locally, partnerships have been established with four Foundry Centres implementing and delivering integrated youth-services (i.e. mental health, substance use, physical health, social services, and peer support). These organizational (national, provincial, community) partnerships will promote mutual project benefits, such that the community-based Foundry centres will be able to better understand and respond to the local needs and preferences of youth. For the provincial and national partners, this partnership will strengthen the development, feasibility, implementation, evaluation and longer-term sustainability of the youth-centred opioid treatment service innovations.

In addition to these partnerships, the ITT project has embedded the co-design process [42, 51] as an instrumental approach to ensure that the needs and ideas of youth, caregivers, and health service providers drive the development and implementation of the opioid treatment service innovations. This process has relevance to increasing interests in the research and practice of person-centred approaches in substance use treatment [56]. Person-centered care (PCC) has been extensively described in the health sciences (see for example, [57–59]). In only the last few years however, it has become a common recommendation to improve the quality of substance use treatment delivery [56, 60, 61]. Much like the elements of the co-design process, PCC puts the unique preferences and needs of the person at the forefront of treatment planning, delivery, and follow-up [56]. This process is enhanced through empathic relationships and shared decision-making [56, 62]. Thus, co-design can be considered a person-centered method that has the potential to guide *how* person-centered practice innovations are developed and implemented, a critical gap that has been identified in this emerging field [56].

## Limitations

Despite the novelty and potential implications of this project to improving youth-centered opioid treatment services, there are limitations that arise from the planned sampling and recruitment activities. First, our sample definition and eligibility criteria of each participant group may be considered broad. This decision was made in light of youths’ poly-substance use profiles [63], transitions in opioid use over time [64], and that best practices recommend multiple forms of treatment that are delivered in diverse settings [14, 15, 25]. Thus, in order to remain sensitive to these patterns and experiences, we followed broader sample definitions. Second, our planned sampling and recruitment strategy relies on the support of community-based partners. This means that we will more easily reach participants who are already engaged in Foundry’s services. While this is beneficial to promoting participants’ safety and comfort during the project, it may affect the breadth of experiences, needs, and solutions pertaining to other settings (e.g. emergency departments, drop-in centres). To strengthen the specificity of our findings, we will explore opioid use profiles (e.g. heroin use vs. non-medical prescription opioids) and treatment settings (e.g. MOUD, detoxification, etc.) where possible in the disaggregated descriptive and thematic analyses.

## Conclusion

To our knowledge, this is one of the first projects to strategically combine the complementary features of CBPR and co-design to understand, develop, and implement youth-centred opioid treatment service innovations. This unique combination of CBPR and co-design means that these innovations will be more effective and readily adopted, as they are driven by the needs and expertise of the multiple stakeholders in this project. Moreover, the evidence generated from this project will respond to outstanding gaps in the literature regarding opioid use treatment needs and solutions from youth, their caregivers, and service providers. Such evidence is critical to informing a pan-Canadian youth-centred approach to opioid use and OUD.

## Supplementary Information

**Additional file 1. **Phase 1 Design, Workshop Activities Schedule and Supporting Materials.

**Additional file 2. **Phase 2 Prototype Selection Activities and Supporting Materials.

## Data Availability

Not applicable.

## Abbreviations

OUD: Opioid use disorder
MOUD: Medications for opioid use disorder
CBPR: Community-based participatory research
BC: British Columbia
ITT: Improving Treatment Together project
CCSA: Canadian Centre on Substance Use and Addiction
PCC: Person-centred care
