# Supplementary material for: Improving Treatment Together: a protocol for a multi-phase, community-based participatory, and co-design project to improve youth opioid treatment service experiences in British Columbia
Source: Addict Sci Clin Pract. 2021 Aug 14;16:53. doi: 10.1186/s13722-021-00261-7 (PMC8364413; doi:10.1186/s13722-021-00261-7)
Supplement: Supplementary file 1 — Additional file 1. Phase 1 Design, Workshop Activities Schedule and Supporting Materials. [file 13722_2021_261_MOESM1_ESM.pdf]

Additional File 1: Phase 1 Design, Workshop Activities Schedule and Supporting Materials

Figure 1. Phase 1 human-centred co-design thinking process (Adapted from Design Council)[1]

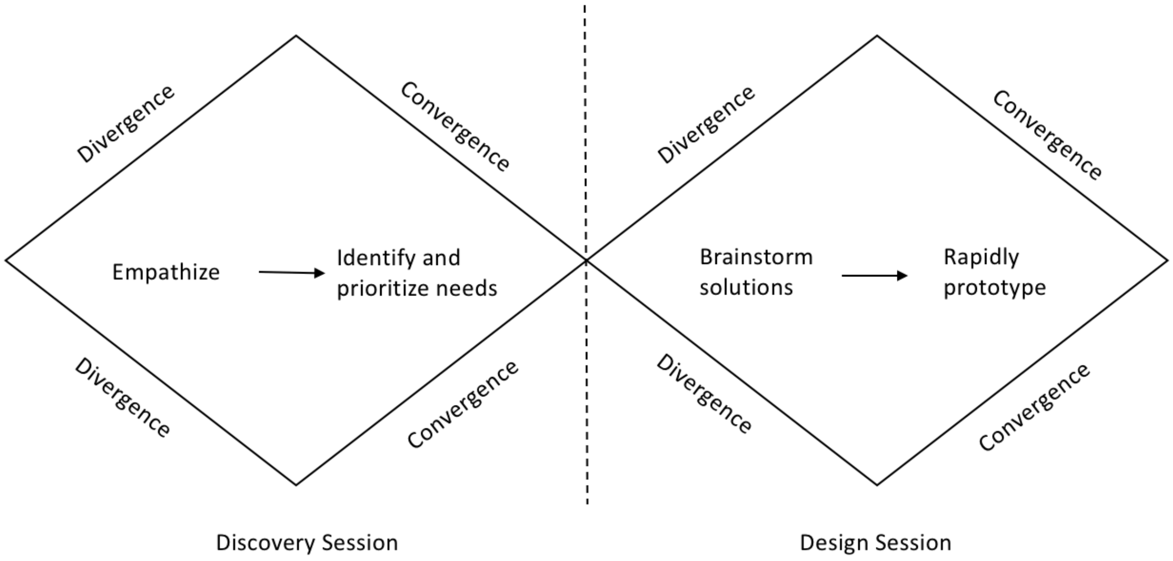

Figure notes:

In the Discovery session, divergent design thinking was used to broadly understand the set of people's experiences with opioid treatment services and to converge these experiences into problem statements around the critical needs to be designed around.

In the Design session, divergent thinking was used to brainstorm as many solutions as possible and then to converge these solutions to a limited number of solutions to be selected for rapid prototyping.

[1] Design Council. The double diamond: A universally accepted depiction of the design process. https://www.designcouncil.org.uk/news-opinion/double-diamond-universally-accepted-depiction-design-process

THE ITT PROJECT
IMPROVING TREATMENT TOGETHER

Figure 2. Phase 1 Workshop Schedule by Session

Discovery Session (3.5 hours) – START TIME: 8:30AM

15 mins	Arrival and individual informed consent
30 mins	Introductions and Creating a Safe Space
45 mins	Understanding experiences: what is it like in your community/context?
15 mins	Break
30 mins	Understanding experiences continued: what is it like in your community/context?
60 mins	Understanding needs: where are the opportunities in your community/context? What are they?
15 mins	Wrap-up

One-hour meal break - catered

Design Session (3.5 Hours) – START TIME: 1:00PM

15 mins	Arrival and individual informed consent (those who missed morning session)
15 mins	Welcome and Introductions
30 mins	Understand the opportunities: Review the findings (and needs) from the Discovery Session
45 mins	Design solutions: Generate ideas for meeting the needs/opportunities
15 mins	Break
75 mins	Design solutions continued: prototype ideas, refine prototype design
15 mins	Wrap-up

THE ITT PROJECT

IMPROVING TREATMENT TOGETHER

Figure 3. Empathy Map Handout ***(Adapted with support and permission from Overlap Associates[2])***

Put yourself into those moments of direct interaction. Map out all of the different elements of those experiences. You can be as conceptual or as literal as you would like but try not to focus on just one specific experience. Try to pick out those things that happen for you across experiences, things that keep coming up.

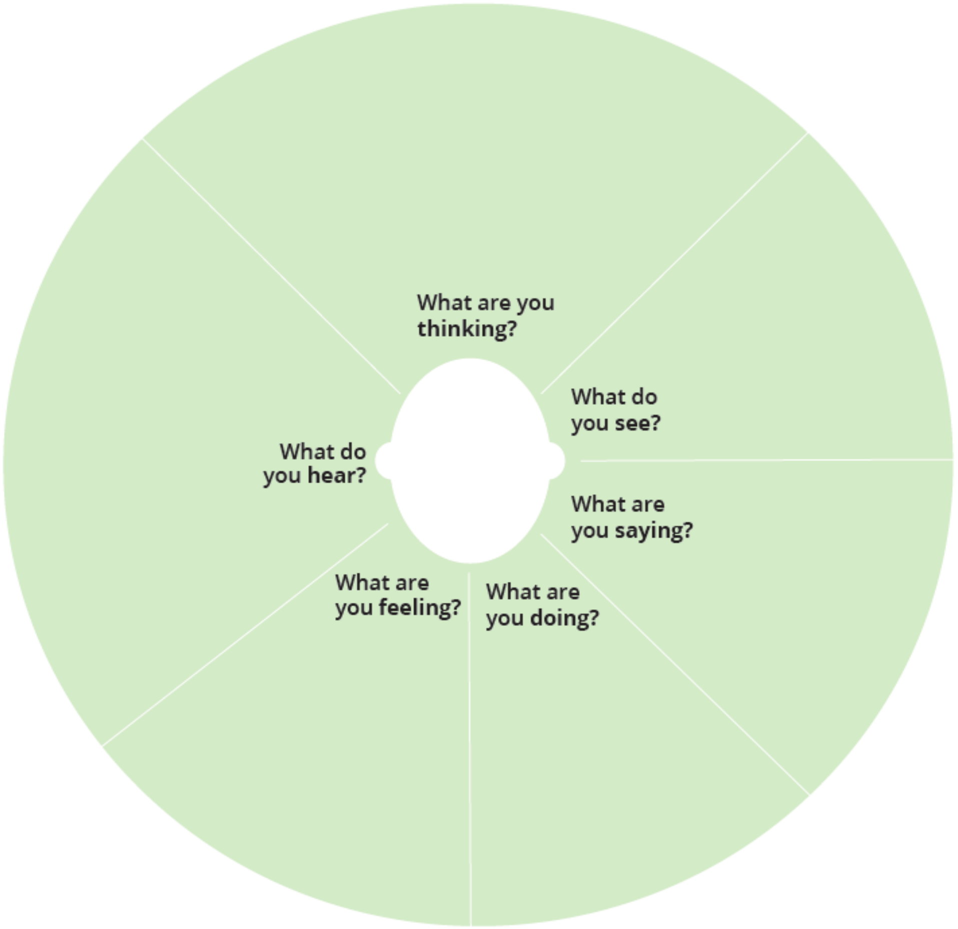

What were you trying to achieve?

1.

2.

3.

What do/did you need to achieve this?

1.

2.

3.

What made it difficult to achieve the above?

1.

2.

3.

[2] Overlap Associates. Empathy map. Kitchener, ON: https://overlapassociates.com/ideas/how-to-use-an-empathy-map/; 2018.

Figure 4. Journey Map Handout *(Adapted with support and permission from Overlap Associates[3])*

At each step:	Step #: _____ Deciding to get help/access services	Step #: _____ Figuring out how/ where to access services	Step #: _____ Recieving services	Step #: ____ After receiving services
What did you expect or hope would happen?				
What actually happened (+/-)?	+	+	+	+
	-	-	-	-
What would have improved the experience?				

---

[3] Overlap Associates. Journey map. Kitchener, ON: https://overlapassociates.com/resources/journey-map/; 2018.

Figure 5. "How Might We …?" Handout ***(Adapted with support and permission from Overlap Associates[4])***

Look back on the individual opportunities and themes that came out of the previous activity. Which ones do you feel really strongly about and would like to see action taken on? Which ones would YOU like to design solutions for? Pick your top 1, 2 or as many as 3 different opportunities or themes and write them below. Then take that opportunity and turn it into a "how might we …. ?" statement.

**Your top choice (or choices):**	**What are some specific, actionable questions that relate directly to the unique and specific opportunity of interest?**
**Opportunity/Theme 1:**	1. How might we….
**Opportunity/Theme 2:**	2. How might we…
**Opportunity/Theme 3:**	3. How might we….

[4] Overlap Associates. Resources. Kitchener, ON: https://overlapassociates.com/resources/; 2018.

Figure 6. Prototype Pitch Handout ***(Adapted with support and permission from Overlap Associates[5])***

Before or while you're creating your prototype, fill out these details about your design idea. Write out the "How might we …?" statement that you designed around, as well as the opportunity or theme(s) that inspired your "How might we …?" statement. List out the impact (or impacts) you hope your design will have (be as specific as possible). Finally, provide a short, written description of your design idea (think about who, what, where, how – once again, try to be specific). This handout will help you deliver your pitch for your prototype in the next activity!

HOW MIGHT WE:

Opportunities/Themes that informed your design:

-
-

Intended impact(s) of your design idea:

-
-

Description of your design idea (what, when, where, how): ____

____

____

____

[5] Overlap Associates. Resources. Kitchener, ON: https://overlapassociates.com/resources/; 2018.
