# Supplementary material for: Improving Treatment Together: a protocol for a multi-phase, community-based participatory, and co-design project to improve youth opioid treatment service experiences in British Columbia
Source: Addict Sci Clin Pract. 2021 Aug 14;16:53. doi: 10.1186/s13722-021-00261-7 (PMC8364413; doi:10.1186/s13722-021-00261-7)
Supplement: Supplementary file 2 — Additional file 2. Phase 2 Prototype Selection Activities and Supporting Materials. [file 13722_2021_261_MOESM2_ESM.pdf]

THE ITT PROJECT

Additional File 2: Phase 2 Prototype Selection

Table 1. Major activities for Phase 2 prototype selection

1. Create community and stakeholder specific prototype summary tables<br>o Highlight similarities between prototypes
2. Collapse table by combining similar prototypes together (can note differences and identify what is most feasible in step 3)
3. Determine which prototypes are feasible based on the following Internal Rubric:<br>o Was the prototype identified by multiple participant groups? At multiple centers?<br>o What is the estimated cost of developing this idea?<br>o Can the idea be developed within our Phase 2 timeline?<br>o Is the prototype scalable to other communities and other populations?<br>o Is the prototype sustainable, such that it can be maintained after the project is completed?<br>o Can this prototype be developed in a virtual context?
4. Conduct an environmental scan to ensure that prototypes are not already under development elsewhere
5. Create a final list of prototypes to present to the community partners and youth team members, including the rationale for choosing each prototype (from step 3)
6. Present and discuss the final list of prototypes to the community partners and youth team members<br>o Send prototypes via email, include External Rubric for selecting prototypes (see Table 2)
7. Hold a prototype decision-making meeting with all of the community partners
8. Share final selection of prototypes with local, provincial and national partners

Table 2. External rubric for selecting prototype

CRITERIA	GUIDING QUESTION
**Potential impact**	If the idea was successful, would it make a key contribution to deepening impact in the community?<br>(1 = low impact; 7= high impact)
	Does the idea have the direct potential to improve treatment for youth experience harms from opioids?<br>(1= indirect potential;7= direct potential)
**Novelty**	How novel is the idea (i.e., does the idea already exist in some form)?<br>(1= already exists;7= new idea)<br>If it already exists in some form, does the prototype have potential for synergy with something that already exists? (Y/N)
**Organizational match**	Is the idea consisted with the strengths and goals of the organization?
	Is the prototype sustainable following the end of the project without additional financial resources?
**TOTAL SCORE**	Add up the scores from the above categories
**Budget required**	What is the estimated cost of developing this idea?
**Timeframe**	Can the idea be developed, testing and evaluated in 9-12 months?
**Impact beyond this project**	Is the prototype scalable to other communities and other populations?<br>(i.e., would other regions or groups benefit from developing this idea??
